# Microcephalic osteodysplastic primordial dwarfism type II is associated with global vascular disease

**DOI:** 10.1186/s13023-021-01852-y

**Published:** 2021-05-20

**Authors:** Angela L. Duker, Dagmar Kinderman, Christy Jordan, Tim Niiler, Carissa M. Baker-Smith, Louise Thompson, David A. Parry, Ricki S. Carroll, Michael B. Bober

**Affiliations:** 1 Skeletal Dysplasia Program, Division of Orthogenetics, Nemours/Alfred I. duPont Hospital for Children, 1600 Rockland Road, Wilmington, DE 19803 USA; 2 Department of Research, Nemours/Alfred I. duPont Hospital for Children, Wilmington, DE USA; 3 Potentials Foundation, Sandoval, IL USA; 4 Gait Laboratory, Nemours/Alfred I. duPont Hospital for Children, Wilmington, DE USA; 5 Department of Cardiology, Nemours/Alfred I. duPont Hospital for Children, Wilmington, DE USA; 6 The South East of Scotland Clinical Genetic Service, Western General Hospital, Edinburgh, UK; 7 MRC Human Genetics Unit, MRC Institute of Genetics and Molecular Medicine, The University of Edinburgh, Edinburgh, UK

**Keywords:** MOPDII, Primordial dwarfism, Vascular disease, Moyamoya, Aneurysm, Stroke, Myocardial infarction, Diabetes, Chronic kidney disease, KaplanMeier

## Abstract

**Background:**

Microcephalic osteodysplastic primordial dwarfism type II (MOPDII) is the most common form of primordial dwarfism, caused by bialleic mutations in the pericentrin gene (*PCNT*). Aside from its classic features, there are multiple associated medical complications, including a well-documented risk of neurovascular disease. Over the past several years, it has become apparent that additional vascular issues, as well as systemic hypertension and kidney disease may also be related to MOPDII. However, the frequency and extent of the vasculopathy was unclear. To help address this question, a vascular substudy was initiated within our Primordial Dwarfism Registry.

**Results:**

Medical records from 47 individuals, living and deceased, ranging in age from 3 to 41years of age were interrogated for this purpose. Of the total group, 64% were diagnosed with moyamoya, intracranial aneurysms, or both. In general, the age at diagnosis for moyamoya was younger than aneurysms, but the risk for neurovascular disease was throughout the shortened lifespan. In addition to neurovascular disease, renal, coronary and external carotid artery involvement are documented. 43% of the total group was diagnosed with hypertension, and 17% had myocardial infarctions. A total of 32% of the entire cohort had some form of chronic kidney disease, with 4% of the total group necessitating a kidney transplant. In addition, 38% had diabetes/insulin resistance. Ages of diagnoses, treatment modalities employed, and location of vasculopathies were notated as available and applicable, as well as frequencies of other comorbidities.

**Conclusions:**

It is now clear that vascular disease in MOPDII is global and screening of the cardiac and renal vessels is warranted along with close monitoring of blood pressure. We recommend a blood pressure of 110/70mmHg as a starting point for an upper limit, especially if the individual has a history of neurovascular disease, chronic kidney disease and/or diabetes. Additionally, providers need to be at high alert for the possibility of myocardial infarctions in young adults with MOPDII, so that appropriate treatment can be initiated promptly in an acute situation.

## Background

Microcephalic osteodysplastic primordial dwarfism type II (MOPDII; OMIM #210720), first described by Majewski, Ranke, and Schinzel (1982) [1], is the most common of the microcephalic primordial dwarfism syndromes [2,4]. MOPDII has autosomal recessive inheritance and is caused by mutations in the pericentrin (*PCNT*) gene [5, 6]. Aside from the classic features of severe pre- and post-natal growth failure together with microcephaly, individuals with MOPDII have characteristic facial features, skeletal dysplasia, abnormal dentition, and can develop insulin resistance as well as truncal obesity [2, 7,11]. There is also a well-documented increased risk for cerebrovascular disease [2, 7, 12,16].

Care has changed dramatically over the past decade for individuals with this diagnosis, and has become more proactive than reactive in some domains, as associated medical conditions became known and screening for these issues could be initiated. With genetic testing now clinically available, a diagnosis can be quickly confirmed and recommended care guidelines put in place, to screen for neurovascular abnormalities, orthopedic manifestations, and insulin resistance [7]. Appropriate charts are also available for monitoring of growth [17, 18].

As neurovascular screening by imaging began to be performed in this population, treatments could be initiated if moyamoya and/or aneurysms were identified, oftentimes before clinically apparent. As children became young adults and survived past these potentially life-limiting neurovascular concerns, new medical complications have emerged including: renal failure necessitating kidney transplantation and coronary artery disease leading to myocardial infarctions [7, 19, 20]. To date, the frequency of these latter issues have not been extensively reviewed in this population. Given this, it is clear that another comprehensive review is necessary at this time, specifically to ascertain the risk of vascular issues of the brain, heart, and kidney, as well as systemic hypertension. The goal is to better understand the landscape of the medical conditions this population will face, so that even more robust proactive screening guidelines and/or medical treatment can pre-empt the life-threatening complications that may arise.

## Methods

To systematically ascertain the medical problems associated with MOPDII and other forms of microcephalic primordial dwarfism (MPD), a Primordial Dwarfism Registry was established in 2008 at the Alfred I. duPont Hospital for Children. The study has been approved by the hospitals ethics board, specifically Institutional Review Board protocol#: 83142. The registry collects retrospective medical records. To date, 128 patients with various forms of MPD have been enrolled via a written consent process. Fifty five of these individuals have a diagnosis of MOPDII.

In 2018, we created a MOPDII Vascular Substudy for those specifically with a clinical and/or molecular diagnosis of MOPDII already within the Primordial Registry. Forty-two families/individuals were able to be re-contacted, and written consent was obtained from 40 families for this specific project; 1 had declined outright after the death of their child, and 1 could not complete the consent process. Of those consented, 6 partially and 24 fully completed a detailed questionnaire on this topic, online in REDCap. Information obtained from the questionnaire was used to document the medical journey of each, as well as acted as guidance for requesting specific medical records of interest, to obtain the most vetted relevant data possible.

Of everyone enrolled in the Primordial Registry with MOPDII, 8 patients were removed from review (75% were international patients) due to lack of sufficient medical records. Therefore, 47 individuals data was used for analysis, see Table 1 for demographic information. If medical records existed in the registry, they were used in this dataset, independent of enrollment status in the substudy. For any valid record releases in the registry, updated records were requested from any known medical institutions, in order to obtain as much up-to-date data as possible for analysis. However, some hospitals would not provide interim information if the record release was not signed within the year, and we had difficulty in re-contacting several families. Ages were calculated and data was collected up to September 15, 2019.

**Table 1:** Demographics

		% (n)	Mean age+/SD (years)	Median age (minmax) (years)
Consented	Registry	100% (47)	15.96+/9.5	14 (341)
	Survey	81% (38)	16.03+/10.1	14 (341)
	Completed	51% (24)	19.7+/9.8	18.5 (341)
	Partially	13% (6)	14.3+/9.9	12 (528)
	Not completed	17% (8)	6.3+/3.8	5 (314)
Sex	Female	47% (22)	16.1+/10.0	13.5 (337)
	Male	53% (25)	15.8+/9.1	14 (341)
Status				
Living	Current age	72% (34)	14.1+/8.9	13 (337)
Deceased	At age	28% (13)	20.8+/9.6	22 (741)
Race		*n*		
White		35		
Asian		5		
Black		2		
Hispanic		2		
Aboriginal		1		
Native Hawaiian		1		
Unknown		1		
Country				
USA		32		
UK		5		
Australia		2		
Canada		2		
Ireland		2		
Netherlands		2		
Guatemala		1		
New Zealand		1		

It is worth noting that sometimes the answers to the questionnaire differed from medical records. For example, for some on a multitude of medications, the questionnaire answer was no to any medical issues, both when provided a list of possibilities and when allowed to enter the answer as free text. In these instances, we defaulted to medical records and past personal communication with the individuals.

Additionally, it became apparent in reading reports that moyamoya is a somewhat subjective radiographic diagnosis, as neurosurgeons had differing opinions on the same imaging. However, once moyamoya was identified by at least one specialist, we notated it as such. There were some instances where a child had a documented ischemic stroke, but was not formally diagnosed with moyamoya until a later date. For the purposes of this analysis, in these instances we denoted the date of moyamoya diagnosis as the date of the ischemic stroke. We noted some discrepancy in aneurysm diagnosis as well. There were instances when an aneurysm was diagnosed, and in hindsight the neurosurgeon could visualize the subtle imaging changes, but the prior image report did not identify that finding. In these instances, we denoted the aneurysm diagnosis as the later date, once it was reported without ambiguity.

Locations were tallied for not only those diagnosed as moyamoya, but also vessels designated as stenotic, narrow, or hypoplastic by brain MRA. The location of any aneurysms, as well as outpouchings also known or tiny aneurysms were included as well. When an individual had many or multiple aneurysms designated in their report, they were only counted as 1 for a particular location, since the exact number was unknown. When stenotic vasculature included multiple branches of an artery and/or different locations, then each branch was tallied with a 1. Additionally, some individuals were only noted to have moyamoya and/or aneurysms, with location unspecified.

Also, some information was not categorizable, so unfortunately not all information could be included in the statistics. For example, a child with a history of both moyamoya and aneurysms was noted to have had neurosurgery in childhood according to parental questionnaire; as it was unknown if this was a moyamoya or aneurysm repair, this surgery was not tabulated.

In order to determine survival probabilities, a KaplanMeier survival analysis was conducted using R (version 3.6.1), the survminer [21] and survival [22] packages. Data included the age of last contact, the status at last contact (alive or deceased), and any associated categorical data, in this case whether individuals had moyamoya and/or intracranial aneurysm(s). Survival probabilities from one time point to the next were computed as P(i)=1-d/n where d is the number who have died since the last time point, and n is the number of remaining individuals. This probability was then multiplied by the prior survivability probability S(i1) so that the current overall survival probability S(i)=P(i)S(i1). When a subject was lost to follow-up or censored, the number of deaths for that time interval was zero, so that the survival curve remains level. Censored data are marked on the curves by vertical bars while deaths are marked by downward steps in the survival function. A log-rank statistic was computed to determine if there was a significant difference in the survival curves in the two groups. Significant differences were considered to occur if the resultant p value was less than 0.05.

## Results

As Table 1 demonstrates, the 47 individuals included in this analysis were composed of 25 males and 22 females, who were primarily white, and from 8 different countries. The living individuals ranged in age from 3 to 37years; median age was 13years. The deceased individuals died at 7 to 41years of age; median age was 22years. Forty four diagnoses were molecularly confirmed with biallelic variants identified in *PCNT* (4 of these by parental report). One individual had a clinical diagnosis of MOPDII with classic features and MOPDII comorbidities, but did not have molecular testing as he died before it was clinically available. The final two individuals did not have biallelic mutations identified, but absence of PCNT protein was observed in a research setting [18].

### Mortality

**Fig. 1:**
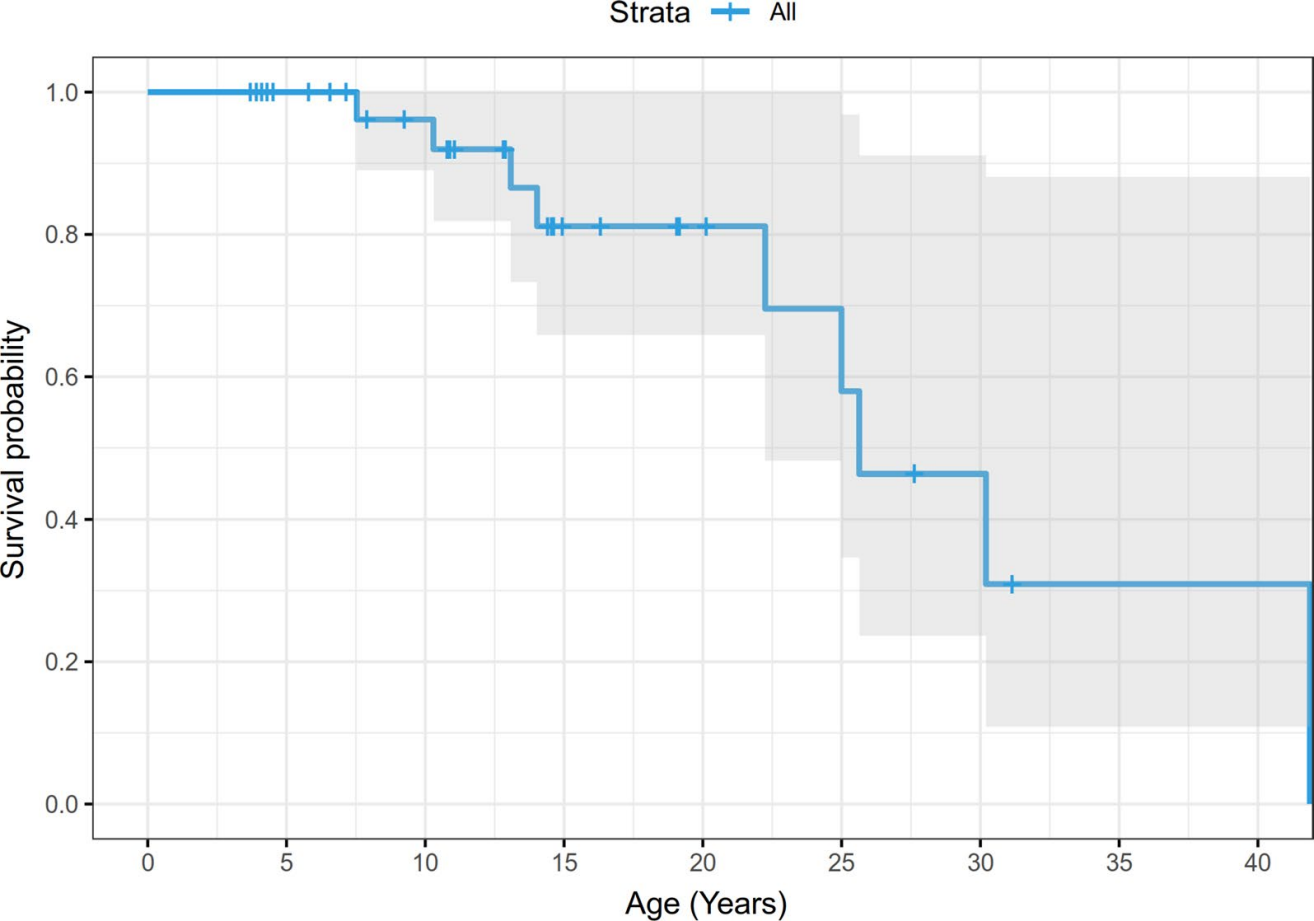
KaplanMeier curve of overall survival, among 47 individuals with MOPDII

A KaplanMeier (KM) survival curve was constructed for this population; see Fig.1. A shortened lifespan can be anticipated with a diagnosis of MOPDII, with mortality predominantly in adulthood. Of the 13 deaths recorded, causes of death include: ruptured brain aneurysm (5 individuals, ages 724years), respiratory failure with acute on chronic renal disease and hypertension (1 individual, age 30years), myocardial infarction/coronary artery disease (3 individuals, ages 1825years), heart/multiorgan failure after surgery (1 individual, age 30years), with 3 of unknown cause (ages 1341years). Two of the unknowns had a history of moyamoya revascularization surgery, aneurysms, stroke, and hypertension (ages 13 and 24years; the elder also had a history of coronary artery disease and prior myocardial infarction); the third unknown cause had no known neurovascular history but did have stage 4 kidney failure with a prior myocardial infarction (age 41years).

### Neurovascular

**Fig. 2:**
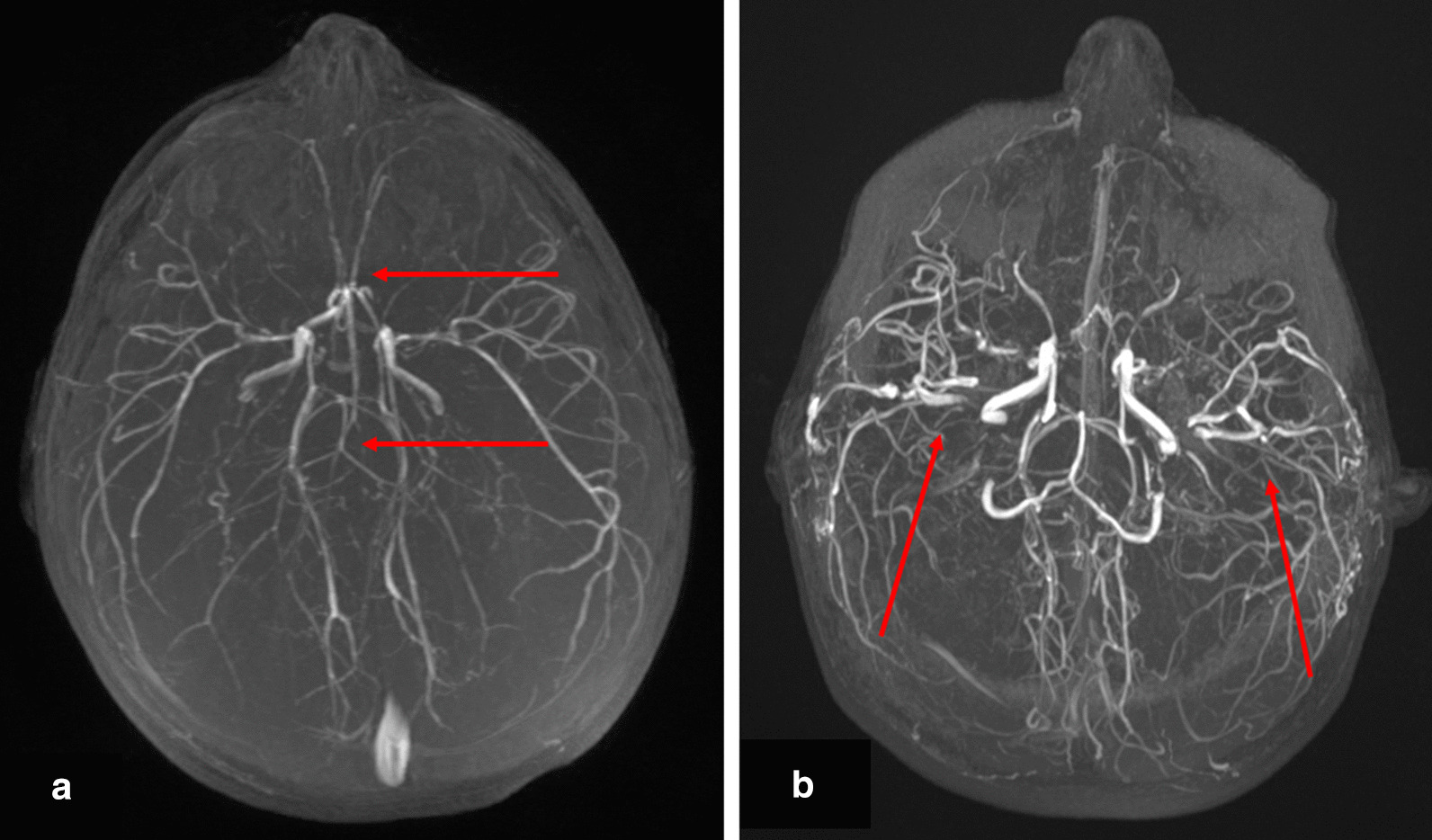
Maximum intensity projection (MIP) of 3-D time-of-flight MR Angiogram (MRA) of the brain of a 23month old female (**a**) demonstrating marked stenosis and collateral development; attenuation of both the anterior and posterior circulation is demonstrated (arrows). MIP of MRA of the same child at 12years of age (**b**), 10years after bilateral pial synangiosis repair; multiple areas of revascularization are now demonstrated (arrows)

In this cohort of 47 individuals, 47% (n=22) were diagnosed with moyamoya (ages 6months24years of age; Table 2), and 19 underwent moyamoya revascularization bypass surgery (ages 7months28years of age; mean: 7.34+/7.1years, median: 4years) by encephaloduroarteriosynangiosis or pial-synangiosis. See Fig.2. A majority (n=15) were documented as bilateral bypasses, but 3 were noted to be unilateral, plus 1 with unknown laterality. One individual had more than one surgical bypass of a side. Also, 13 individuals had stenotic or hypoplastic neurovasculature that was never ultimately diagnosed as moyamoya.

**Table 2:** Comorbidities

	% of total (n)	Mean age+/SD (Years)	Median (minmax) (years)
Neurovascular diagnoses			
Moyamoya	47% (22)	6.6+/6.8	3 (024)
Intracranial aneurysms	53% (25)	11.9+/8.0	9.5 (237)
Both	36% (17)	9.3+/5.9	9 (218)
Neither	36% (17)	13.4+/11.2	12 (341)
Other co-morbidities			
Hypertension/prehypertension	49% (23)	14.7+/8.0	13 (036)
Diabetes/insulin resistance	38% (18)	13+/4.6	11 (826)
Anemia	79% (37)	6.8+/8.3	2 (026)
Thrombocytosis	79% (37)	5.3+/7.3	2 (026)
Hypercholesterolemia	32% (15)	18+/7.6	18 (536)

Regarding brain aneurysms, in this total cohort of 47 individuals, 53% (n=25) had aneurysms identified (at 237years of age; Table 2). Sixteen individuals had more than one aneurysm detected, including 5 individuals with an uncountable multitude of aneurysms/outpouchings per radiology reports. When specifically listed, some individuals had more than 10 aneurysms identified by MRA imaging. Fourteen individuals underwent aneurysm obliteration by clipping, coiling or stenting (at 937years of age; mean: 15.2+/7.1years, median: 14.5years). Eight have had more than one aneurysm surgery; the highest number of surgeries for aneurysm treatment we had documented by any one individual was 4. One individual additionally had a 3mm left middle cerebral artery aneurysm treated after moyamoya revascularization; it was never again visualized on subsequent imaging.

**Fig. 3:**
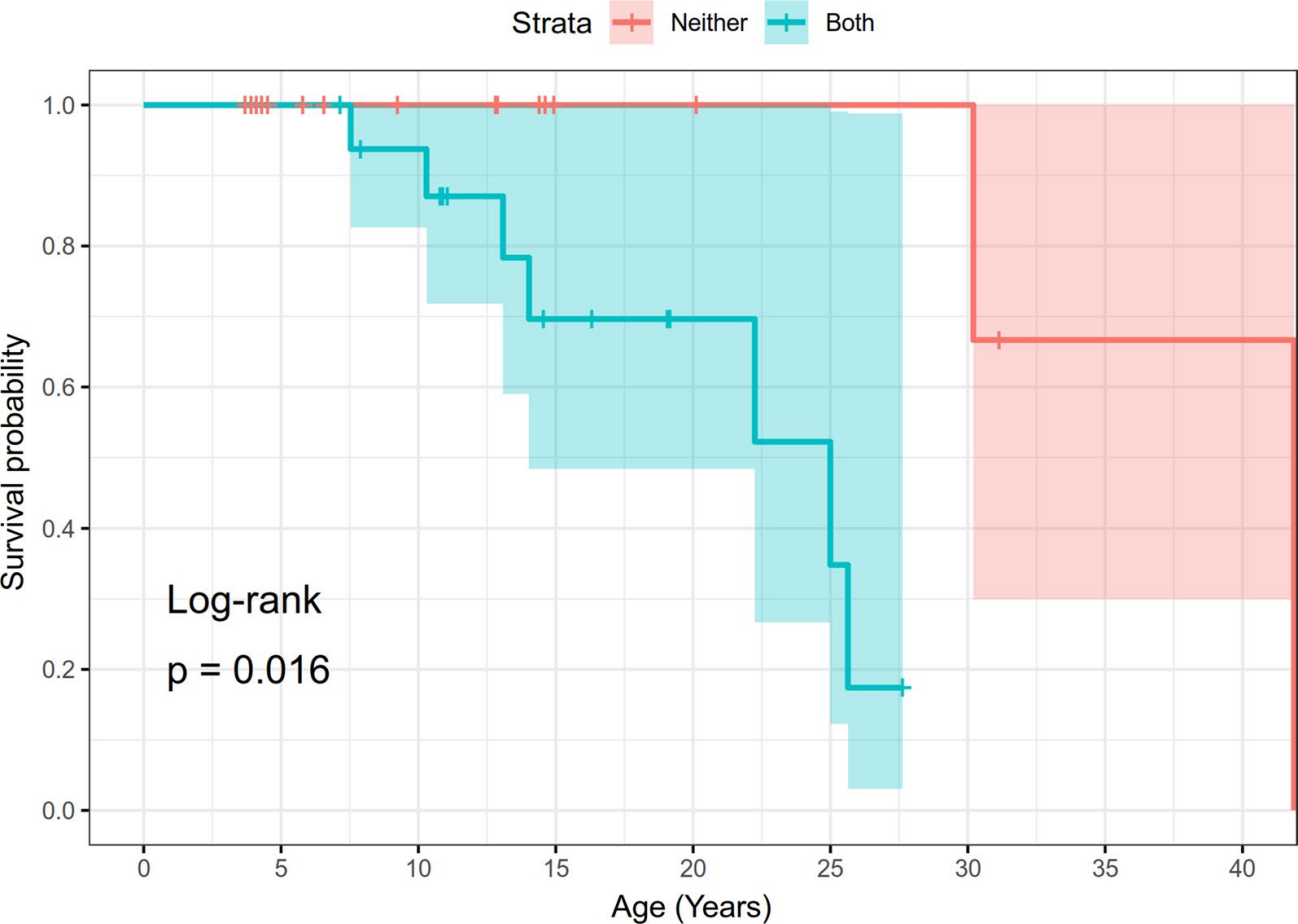
KaplanMeier analysis of survival, according to neurovascular disease status, among 47 individuals with MOPDII. Both signifies diagnoses of both moyamoya and intracranial aneurysms, and neither denotes individuals with no known neurovascular disease

A KaplanMeier survival curve was additionally constructed by stratifying for neurovascular disease status, specifically diagnoses of intracranial aneurysms and moyamoya (both vs neither diagnosis); see Fig.3. These diagnoses contribute to the morbidity and mortality of this condition. Of all the individuals with neurovascular diagnoses, 15 were known to have an ischemic stroke, with an additional 3 individuals noted to have TIAs or events with TIA-like symptoms. Also, 11 individuals were noted to have a hemorrhagic stroke, 3 of whom had more than 1 hemorrhagic stroke. Overall, six individuals with ischemic strokes also had a history of hemorrhagic strokes, and one individual with TIAs also had a history of hemorrhagic stroke. Of everyone with a diagnosis of moyamoya, 55% had documented ischemic strokes, and 44% of individuals with aneurysms had a hemorrhagic stroke.

Not everyone with MOPDII has one of these neurovascular diagnoses. There were 17 individuals (36%) diagnosed with both moyamoya and intracranial aneurysms, but 17 individuals (36%) with neither; Table 2.

**Fig. 4:**
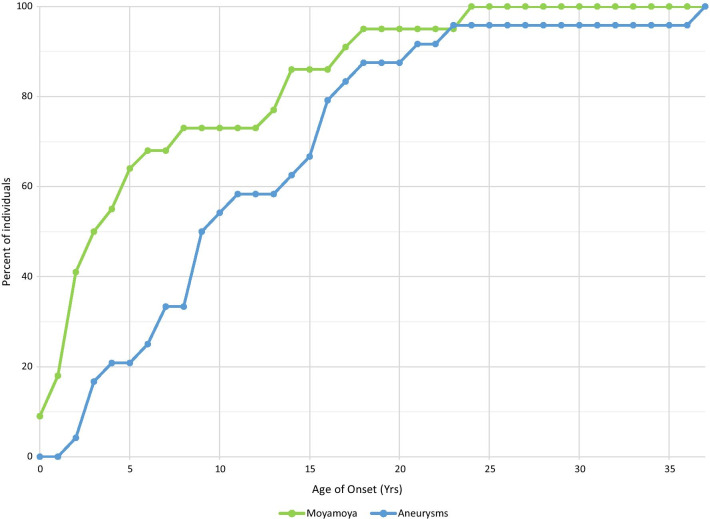
Moyamoya and aneurysms by age at diagnosis

In general, the age of diagnosis for moyamoya is at an earlier age than aneurysms. Aneurysmal risk appears to increase at a relatively steady rate throughout childhood, and continues into adulthood, whereas the moyamoya slope is much steeper prior to age 5 (Fig.4). About a quarter of the individuals with moyamoya were diagnosed at older than 8years of age, but each had their own caveats. Specifically: a 13, 17 and 24year old were all diagnosed with moyamoya on their first brain MRA, so it is unclear how long the moyamoya was present. In addition, there was a child diagnosed at 13years of age after numbness with a TIA had a normal MRA 6years prior, but no imaging in between; there was also a child diagnosed at 14years old who had a brain MRA 6years prior that was suggestive but not definitive for moyamoya; lastly, an 18year old had a history of multiple aneurysms, with narrowing and dysplastic vasculature noted on a brain MRA and angiogram 5years before moyamoya appeared in her chart, over time, details were difficult to see in images due to artifact from an aneurysm clip.

Table 3 illustrates the site of neurovascular abnormalities in our cohort of individuals with MOPDII. Overall, of the 35 individuals with moyamoya or a stenosis/hypoplasia of neurovasculature, 37% (n=13) had only the anterior circulation affected, and 6% (n=2) had only the posterior circulation, with 49% (n=17) having both anterior and posterior vasculature involved, and 8% (n=3) with an unknown location affected. Given the complex/compound nature of vessels, one persons stenotic vasculature potentially encompassed multiple vessels and areas of circulation. However, the data point to anterior circulation being more involved with moyamoya in this population. It is also apparent that proximal segments are more affected. That difference is not seen with aneurysms. There is additionally no clear left to right differences noted with either.

**Table 3:** Location of vasculopathies

	Stenotic vasculature			Aneurysms/"outpouchings" ruptured					
	U	R	L	U	R	L	U	R	L
*Anterior circulation*									
ICA									
Infraclinoid						1			
Cervical		7	6						
Petrous		3	3		1				
Cavernous		2	2		2	2			
Superior hypophyseal artery					1				
Supraclinoid/cerebral ICA		16	17		3	2		1	
Ophthalmic		1			1	1			
Anterior choroidal artery	1				1				
NOS		3	3						
ACA									
A1		16	13		1				
A2		3	3	1	2	3			
A3		1							
Pericallosal artery					1	1			
NOS	1		3		1	1			
MCA									
M1		14	16		2	3			
Anterior temporal artery					1				
M2		6	6		1	1			
M3			1						
M>3			1						
NOS	1	2	2		4	5			
*Posterior circulation*									
PCA									
P1		4	4			1			
P2			1		1	1			
P3					1				
NOS		1	2		3	4			1
Basilar	11			6			2		
AICA					1	2			
Vertebral	1	5	6	1	1	1			
PICA		1			3	3		1	1
*Circle of Willis*									
ACOM				4	4		2		
PCOM	2	4	3		6	5			
*External carotid art*			1						
Superficial temporal artery	1	1	1	1					
*Renal artery*									
Upper pole					1				
Mid pole						1			
Unknown location	1								

*U*site unclear or vessel without laterality

*R*right side

*L*left side

### Cardiac

Of the 47 total individuals, 17% (n=8) had premature myocardial infarctions (at 1733years old, mean age: 24+/5.2years, median age: 24years). These included: 3 documented STEMI (ST-elevation myocardial infarction), 1 NSTEMI (Non-ST-elevation myocardial infarction) and 4 with no medical details about their specific cardiac event available. Of the total eight individuals, three experienced more than one acute coronary syndrome event. One additional individual was diagnosed with coronary artery disease (CAD), but did not have a known history of a myocardial infarction.

Of these individuals with CAD/MI, 78% (7/9) were managed with cardiac surgery/catheterizations; the others may have been, but all records were not available to us. These included four with stenting of coronary arteries, two catheterizations without stenting (given extremely small vessel size for one, and because the coronary artery disease was distal for the other), and one with unknown details. Two individuals had more than one cardiac catheterization, and one individual necessitated coronary bypass surgery.

Twenty eight percent (n=13) had a history of ASD, VSD or PFO beyond the newborn period, and one additional individual had multiple rhabdomyomas.

### Renal

Two percent (n=1) were diagnosed with renal artery stenosis; 7 others also had this suggested by renal ultrasound with Doppler, but subsequent MRA/CTA failed to confirm the diagnosis. In addition, 4% (n=2) were diagnosed with renal aneurysms (mean age of 10years); one had the aforementioned renal artery stenosis and went on to have partial infarct of a kidney, and the other individual subsequently had a kidney transplant. See Table 3 for locations.

Eight individuals have been diagnosed with chronic kidney disease, stage III or higher, at a mean age of 22.6years old. Seven additional individuals had record of CKD stage I, II, or unknown stage, to total 32% of the entire cohort with some form of chronic kidney disease. Two individuals underwent kidney transplants, at 23y7m and 21y2m of age.

Thirteen percent (n=6) were noted to have nephrolithiasis (at 1925years of age; mean 21.8years), all of whom were on anti-hypertensive medications. One needed treatment by ureter stenting at 21years of age.

**Fig. 5:**
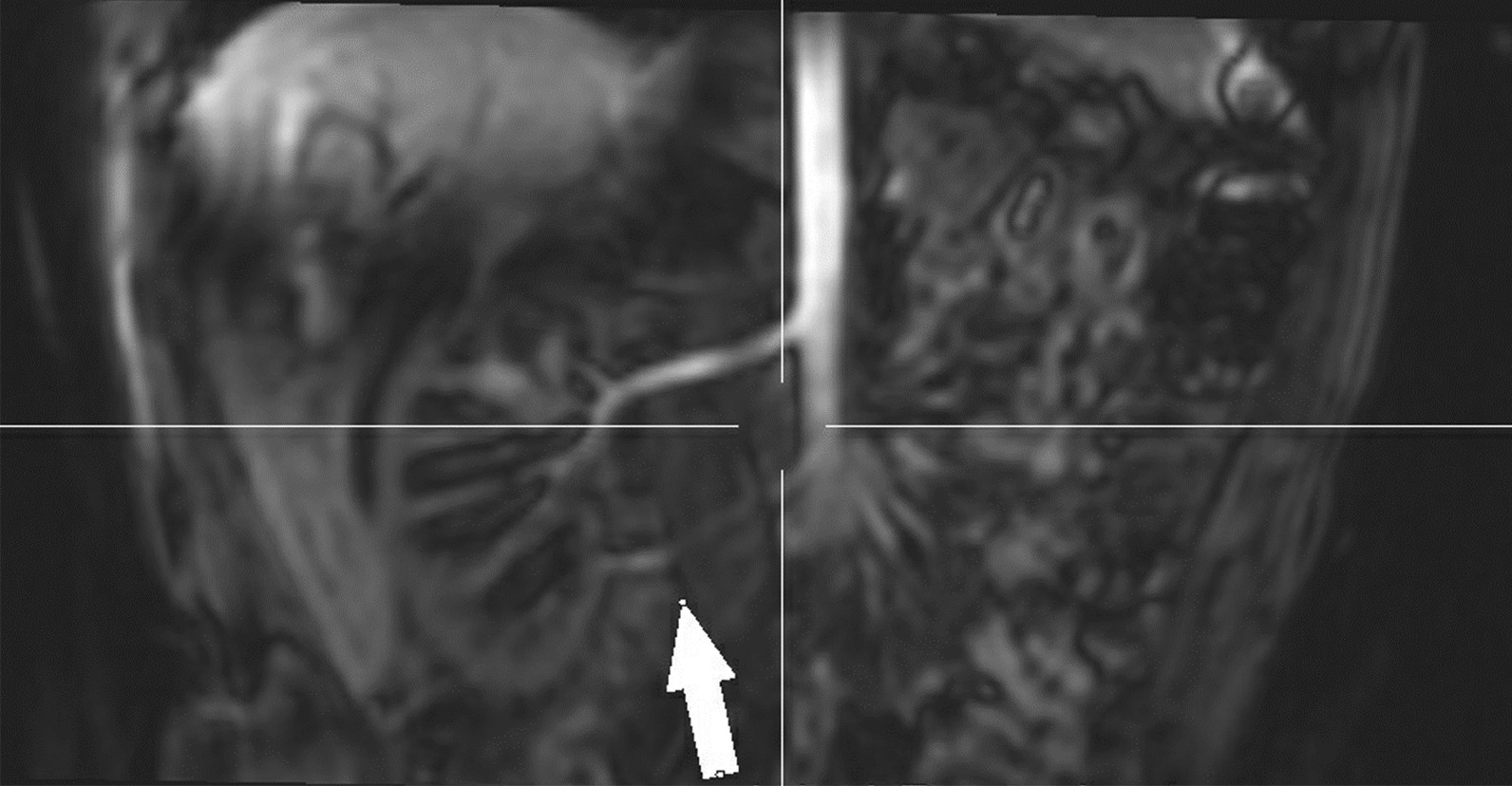
MR Angiogram of the abdomen of a 6year old male, demonstrating an accessory renal artery to the lower pole of the right kidney (arrow)

Regarding structural differences, 28% of males (n=7) had accessory/duplicated renal arteries (Fig.5); interestingly no females had this finding. Additionally, 8% of males (n=2) had hypospadias, and 44% of males (n=11) had cryptorchidism or retractile testes. Six percent of the whole cohort (n=3) were noted to have hydronephrosis.

### Other Vasculopathies

**Fig. 6:**
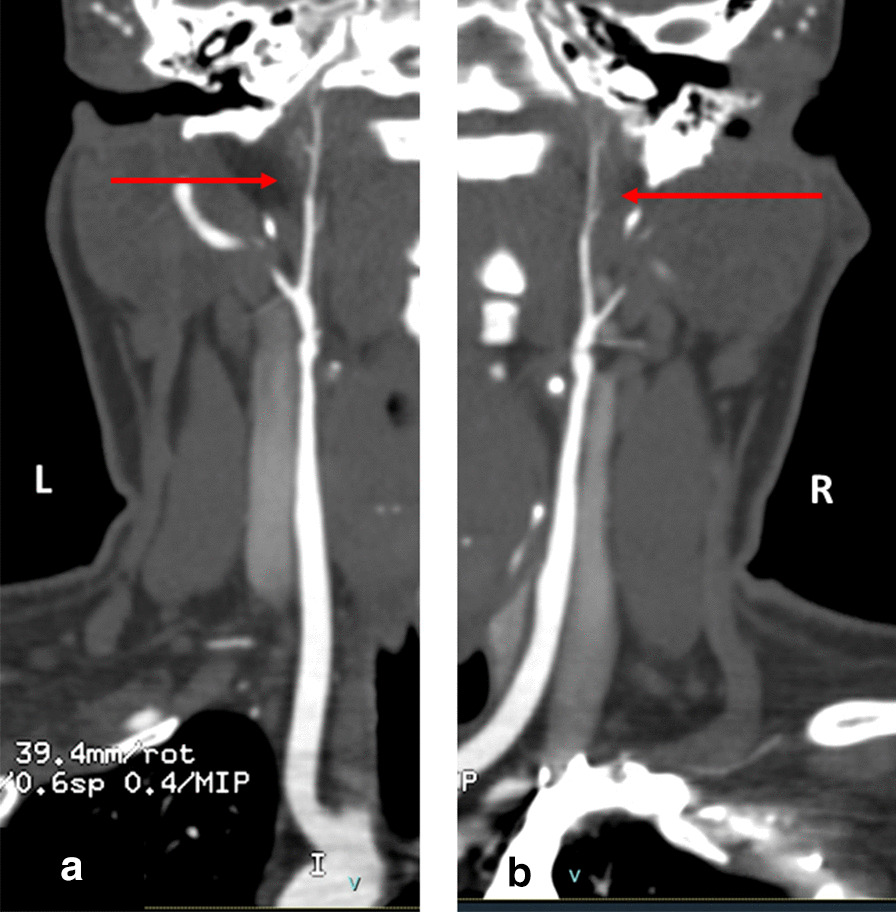
CT Angiogram of the neck with coronal reconstruction along course of the left (**a**) and right (**b**) carotid arteries of a 25year old female. There is marked stenosis of the bilateral internal carotid arteries (arrows) beginning distal to the carotid bifurcation

Beyond that noted in the neurovasculature, renal and coronary vessels, there have been reports of other vascular issues for individuals with MOPDII. Specifically, the external carotid artery was identified as stenotic for 3 individuals (Fig.6), and the site of an aneurysm for one individual (Table 3).

Additionally, the femoral artery has been a common location for insult, given the catheterizations needed for examination and treatment of associated medical concerns. This has occurred for 13% (n=6) of all individuals in our cohort. Three individuals were documented to have femoral artery occlusion after procedures which then necessitated fasciotomies and thrombectomies to treat; however, one went on to need patch and bypass, and after subsequent complications eventually lost the artery and full use of the leg. Another individual was found to have collapse of a superficial femoral artery, treated by bypass; later noted to have thrombosis with subsequent restenosis and revision. An additional individual was noted to have a femoral artery pseudoaneurysm, treated by thrombin injection. Lastly, yet another individual had focal severe stenosis identified in the common iliac artery, leading to stent placement, and subsequent in-stent restenosis.

### Blood pressure

Forty three percent of individuals (n=20) in this cohort had either diagnosed hypertension and/or were treated with anti-hypertensive medications. Another 6% (n=3) were diagnosed with borderline or pre-hypertension; this was the terminology found in their medical records to describe their elevated blood pressure. Among those diagnosed with hypertension and/or elevated blood pressure, the median age was 13years (range 036years old); see Table 2. In addition to recommended lifestyle intervention (e.g., lower salt diet), treatment of hypertensive patients more commonly included: calcium channel blockers (CCB), angiotensin converting enzyme inhibitors (ACEi), and/or beta blockers (BB). In addition, central alpha agonists (e.g., clonidine), vasodilators (e.g., hydralazine), angiotensin II receptor blockers, and diuretics were also recorded.

Criteria for diagnosing hypertension was not always consistent among the population. While some providers chose the convention of defining appropriate blood pressure based upon height percentile, others chose to utilize a set cut-off value such as 130/80. It is evident that anti-hypertensive therapy was not always started when a patient met criteria for hypertension, suggestive of therapeutic inertia in this population.

Given the sporadic and sometimes incomplete nature of data received from some individuals, we are unable to report by percentile according to height, so instead tabulated data by the pressures themselves. The average adult height of males and females with MOPDII is approximately 100cm [18]. According to Flynn et al. [23], a blood pressure of 110/70 approximately corresponds to the 95^th^ percentile for males and females at this height, thus we used that as our cut-off measurement for the analysis.

Seventy two percent (n=34) had at least one documented blood pressure over 110/70mmHg (ages 024years of age; mean: 8.6+/7.5years, median 7years), with 66% (n=31) having a documented blood pressure higher than 120/80mmHg (ages 028years of age; mean: 11+/7.7years, median: 11years), and 47% (n=22) having a documented blood pressure higher than 130/80 (ages 036years of age; mean: 15.5+/8years, median: 15years). A majority of individuals in each blood pressure range had an elevated measurement recorded on at least 3 separate occasions.

**Fig. 7:**
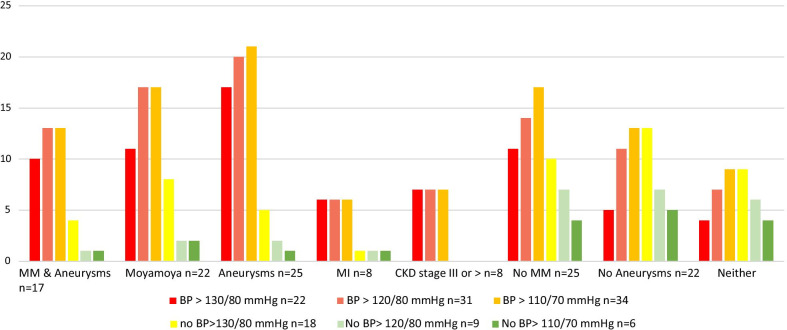
Prevalence of blood pressure with comorbidities

As Fig.7 demonstrates, elevated blood pressure was associated with neurovascular, cardiac, and renal disease in individuals with MOPDII. Measurements greater than 110/70mmHg, were recorded in a majority of individuals with a history of moyamoya (n=17, 77%), aneurysms (n=21, 84%), myocardial infarctions (n=6, 75%), and chronic kidney disease at stage 3 or greater (n=7, 88%). These are likely underestimates, as some included individuals had a prior diagnosis of hypertension but no blood pressure measurements for analysis (which would elevate the CKD percentage to 100% for example), and/or no blood pressure/hypertension information was available for some individuals at all. Additionally, all eighteen individuals with LVH and all eight individuals with dilated aortic root had diagnosed hypertension and/or record of BP>110/70mmHg.

### Comorbidities

Table 2 delineates the frequency of other comorbidities in the total cohort, as well as ages at diagnosis when known. Ninety three percent of individuals (37 of 40 with results) were noted to have laboratory results consistent with anemia (defined as hemoglobin<12g/dL) on at least one occasion. High platelets were also noted in this cohort, with 90% of individuals (37 of 41 with results) having laboratory results consistent with thrombocytosis. The highest platelet count recorded was 180310^9^/liter, which occurred in an individual with both MOPDII and Factor V Leiden.

Fifty eight percent of individuals (15 of 26 with results) had hypercholesterolemia (defined as cholesterol>200mg/dl), at a mean age of 18years old; 13 of these individuals were treated with various statins. The two not noted to be under treatment were the youngest, with hypercholesterolemia detected at 5 and 6years of age.

Insulin resistance was recorded for 39% (18 of 46 with results), including some with frank diabetes. Mean diagnostic age of insulin resistance was 12years, and mean age of type II diabetes diagnosis was 14years of age. Treatment included: metformin, insulin, pioglitazone, linagliptin, ezetimibe, and exenatide, with the majority treated by metformin and/or insulin.

## Discussion

Our primordial registry was conceived over a decade ago out of the hope from many families with MOPDII, that by gathering data together, we would be able to better understand why there did not seem to be any older adults with this diagnosis. Hall et al*.*s exhaustive literature review in 2004 [2] noted the existence of neurovascular anomalies in those with this clinical diagnosis, but many details were unknown at that time; for example, it was unclear if it was congenital or progressive, but it seemed at that time to be accelerated by puberty. Her groups recommendation was to start monitoring for CNS vascular anomalies at least by mid-childhood. Now, about a decade and a half later, MOPDII neurovascular screening guidelines have been more precisely honed, with screening starting at diagnosis [4, 14, 24]. The data presented in this cohort should help expand vascular screening guidelines for MOPDII yet further.

The questionnaire for our MOPDII vascular substudy was complicated, given the details needed to make this review as robust as possible, and the medical complexity of this condition for some individuals. We are surmising that it is for this reason that only 63% of families who consented for the substudy completed the survey in its entirety, as the duration of time needed for some families would be lengthy. A few of the families who did not complete the survey likely did not because they were clinic patients of ours, and we already are well-versed in their clinical course. However, there were also rather medically involved individuals for whom we were left with minimal information. Some individuals enrolled in the Primordial Registry were not included in this studys analysis, as no medical information was known besides their MOPDII diagnosis; we did not want to skew the results due to an inflated denominator. However, we posit that the data that remains is still the most comprehensive compendium of its kind for individuals with MOPDII.

Through presymptomatic screening as well as with imaging performed after clinical signs present, our data demonstrate that about two thirds of individuals developed neurovascular disease, with half of all individuals developing intracranial aneurysms, approximately half identified with moyamoya, and a third with both diagnosed (Table 2). Our results also underscore that moyamoya presents at younger ages than aneurysmal disease (Fig.4), and risk of neurovascular disease continues into adulthood, therefore identification and treatment of one issue should not obviate the need of that individual for future screening [7, 16, 24]. Surgical outcomes for revascularization surgery and aneurysm treatment in MOPDII have been previously described [16].

As our current data show, MOPDII is also associated with vasculopathies beyond the neurovasculature. In our relatively large cohort of an exceedingly rare disease, renal artery stenosis, renal aneurysms, carotid and coronary artery disease were noted. These have not been benign processes; individuals with these findings have gone on to have kidney infarcts, kidney transplantation, and/or myocardial infarctions. Therefore, cardiac and renal screening appears warranted in this population. Although, the precise nature and frequency of screening is not yet fully developed.

This association is not isolated to MOPDII. Diffuse vascular disease has been identified in other genetic conditions, such as those associated with ACTA2 [25], YY1AP1 [26], and NF1 [27, 28]. This association is additionally noted in the general population. On retrospective review, symptomatic coronary disease was noted in 5% of individuals with idiopathic moyamoya disease in a Korean population, where the population frequency of coronary artery disease is less than 1% [29]. There have also been published cases of individuals with moyamoya and peripheral pulmonary artery stenosis with post-stenotic aneurysms [30]. Additionally, renal artery involvement has been noted in 58% of multiple series of individuals with moyamoya in Japan and the Republic of Korea, and a renal artery evaluation has been recommended in general for individuals who have moyamoya concomitant with hypertension [31,33].

Diabetes and insulin resistance are additional important contributing factors, with over a third of individuals in our cohort with one of those diagnoses. (Further detailed study of this association with MOPDII can be found in Huang-Doran et al*.,* 2011 [9].) These alone are associated with both microvascular and macrovascular disease, leading to endothelial dysfunction, smooth muscle cell dysfunction, and platelet dysfunction, with coronary artery disease as the most common cause of death for people with type 2 diabetes [9, 34]. Since dyslipidemia and hypertension are also associated with type 2 diabetes [23, 34], screening of these would also be important for individuals with MOPDII, as they all contribute to cardiovascular complications [9].

Fourteen individuals in this cohort trialed human growth hormone replacement. Some stopped after a few months, and others continued for years. Of these, 71% (10) developed insulin resistance and/or frank diabetes later in life. While true that eight other individuals developed insulin resistance in the absence of growth hormone supplementation, given the published added risks and inefficacy of this treatment [9], would recommend not pursuing growth hormone treatment in this condition.

For the individuals with MOPDII in this study, chronic kidney disease (CKD) appeared independent from renal vascular disease, and is at a frequency that warrants screening as well. CKD itself is associated with a high risk of coronary artery disease, with an elevated risk of cardiovascular mortality beyond that associated with often concomitant hypertension and diabetes [35]. Of our cohort, there were only 3 individuals who were treated for hypertension but were not diagnosed with moyamoya or intracranial aneurysms; two of these three were diagnosed with stage IV CKD and are now deceased. CKD is also known to modify the presentation of coronary artery disease and myocardial infarctions, such that one needs to be critical of atypical symptoms [35]. This is all the more crucial information for individuals with MOPDII, as the current study has documented heart attacks occurring in young adulthood for multiple individuals. Emergency providers need to be at high alert for this possibility so that appropriate treatment can be initiated promptly in an acute situation.

In our MOPDII cohort, approximately half of all the individuals have been diagnosed with hypertension or pre-hypertension. More specifically, one of these clinical diagnoses were recorded in 64% of individuals with a history of moyamoya, 76% with a history of intracranial aneurysms, 88% with a history of myocardial infarctions, and 100% with a history of chronic kidney disease (stage 3 or higher). These are not insignificant numbers, and as we posit above, the true proportions are possibly higher than this since providers are likely under-diagnosing hypertension in this population.

There are recommendations to monitor blood pressure in MOPDII [7, 12, 15], though normal ranges for individuals with this degree of extreme short stature have never been delineated. Using typical sex and age-related norms for individuals who have short stature can lead to misclassification of hypertension as normal [36]. It has been hypothesized that chronic increased pressure of even a moderate amount could be proportionally more damaging to smaller vessels and organs, and that chronic hemodynamic stress on abnormal vessels could drive cerebral aneurysms [7, 14, 15, 24, 37, 38]. It is additionally problematic to modulate blood pressure in individuals with risk of both moyamoya and aneurysms. Anesthesia can be tenuous in these scenarios, as one cannot drop the pressure too low or let it get too high without risk of ischemic or hemorrhagic strokes [15, 39, 40]. The difficulty in monitoring and treatment of hypertension in those with extreme short stature along with a risk of neurovascular disease is what should the top systolic and diastolic pressures be? Besides not knowing the hypertensive range for this population, the difficulty in monitoring is compounded by the challenge of getting an accurate measurement on individuals with such small and slender limbs; oftentimes a neonatal cuff is needed for school age children and younger with MOPDII.

From what we could deduce with the current data, 110/70mmHg should be a good starting point as an upper limit for blood pressure, monitored at each medical encounter, especially if the individual has a history of neurovascular disease, chronic kidney disease and/or diabetes. For individuals with height dramatically less than the mean for MOPDII, blood pressure should likely be lower yet. This is an area that needs further in-depth study. We provide this only as a starting point, as we implore medical providers to be aware of the complexities involved for this population. Untreated hypertension can have catastrophic effects at young ages for those with MOPDII, so should be monitored closely. Also, it is important to not overlook that acute hypertension should be investigated by imaging before treatment, because the etiology could be neurovascular disease or vascular disease elsewhere.

As there are so many variables involved, it may be too difficult to completely tease things out at this juncture. Given the timeline of events and discovery of the natural history of disease, todays adults with MOPDII were getting their first presymptomatic screening for neurovascular concerns in adolescence; many finding treatable conditions at first image. A majority of the individuals who had myocardial infarctions as young adults had moyamoya diagnosed as teenagers or later, and did not have neurovascular imaging to screen starting in infancy as many children now do. This group of individuals likely had chronic hypertension for years without identification and treatment, which certainly could contribute to all the subsequent sequelae of medical complications that are now evident.

Children with MOPDII entering adolescence right now may have a different natural history of disease, as one variable should be eliminated if they have had access to brain MRA or CTA modalities at birth and onward, given the recommendations for neurovasculature screening starting at diagnosis for moyamoya and aneurysms published a decade ago. However, even if neurovascular disease could be detected presymptomatically, and fully treated without sequelae, that is likely not the end of the story as there are multiple people with MOPDII who have had coronary artery disease or chronic kidney disease along with normal neurovascular screens.

### Limitations

As the registry has been in existence for over 10years, many families had consented into the original project, but had stopped proactively sending medical records into the repository. Updated consents would be signed over the years, to allow us to reach out to their treating physicians for records as needed. There were some individuals that died between enrollment in the registry and this substudy. Given the nature of this project, those would be the most important records to identify and obtain. However, considering the sensitive nature of the circumstance, multiple of these families were either not responding to our inquiry, or once contacted, were not interested in participating in the survey or providing updated record releases. For these families we are only aware tangentially of what occurred for their child, which was not included in the larger analysis because the finer details could not be obtained.

## Conclusions

This data underscores the high burden of vascular concerns in this population. Though screening regimes have been published and put in place worldwide for neurovascular issues such as moyamoya and intracranial aneurysms, there are no evidence-based guidelines for screening for the other vascular manifestations. There has also not yet been a focused study on hypertension in this population, which would be an important avenue for further research.

To this end, we recommend the following care guidelines for individuals with MOPDII:

### Neurovascular

As previously published, to screen for neurovascular disease, recommend brain MRA/I at diagnosis and every 1218months thereafter in childhood [7, 14]. This interval was chosen following the observation of a child progress from a normal MRI to stroke in a 2year period of time. If moyamoya is detected and treatment is initiated, we believe that further monitoring for moyamoya be done at the discretion of the treating physician. Screening for aneurysms, however, should continue despite identification and treatment of moyamoya, due to continued risk of development of aneurysms into adulthood; screening should be at least every 2years after the age of 18 [7].

### Renal

Due to structural abnormalities noted above, recommend renal ultrasound at diagnosis. Also, because chronic kidney disease appears independent from renal vascular disease, recommend assessments of renal function starting at age 5 [7]. The most precise way to monitor renal function and glomerular filtration rates is not clear, but looking only for elevated creatinine levels will prove inadequate given the stature and lean muscle mass of this population. Nephrologists following this cohort have used testing such as cystatin c level and inulin clearance to assess and monitor glomerular filtration rates. Additionally, because kidney stones were noted in individuals on antihypertensive medicines, would recommend monitoring for that complication as well for individuals in that situation.

### Cardiac/HTN

Given structural abnormalities noted, echocardiogram is recommended at diagnosis. Blood pressure should be monitored annually [7], with a cuff of appropriate size. For children, the height equivalent 95th percentile pressure limits from Flynn, et al. [23] can be used. Blood pressure should be considered hypertensive if over 110/70 for most adults with MOPDII. For some of shorter stature than average for MOPDII, a lower number could be their cut-off, but 120/80 should certainly not be considered normal for anyone with MOPDII, even in adulthood. Until more precise recommendations can be implemented, we are recommending subspecialist monitoring and care for the management of blood pressure, typically by a nephrologist and/or cardiologist with expertise in hypertension management. Consideration should be given to the use of echocardiogram and ECG for signs of hypertension as well. Although blood pressure measurements should begin at diagnosis and continue during routine check-ups, if non-elevated pressures are found, the referral to subspecialists can likely be deferred until 510years of age. If elevated blood pressures are noted, and before hypertension is treated with medication, due diligence should be performed to rule out the many possible etiologies associated with MOPDII, specifically moyamoya disease as well as renal artery stenosis.

Hypercholesterolemia is another comorbidity which should be screened for and treated appropriately once detected.

Though not specifically studied here, lifestyle interventions associated with lower blood pressure could certainly be considered to minimize other contributing factors, to include: DASH diet, avoidance of high sodium foods, physical activity, and weight management [23].

It cannot be underscored enough that any signs of myocardial infarction (atypical or otherwise) should be taken seriously, given the relatively high incidence at a young age in this population.

Additionally, when performing any needed catheterizations, extreme care should be taken, given the known postoperative complications with femoral artery stenosis.

### Diabetes/insulin resistance

Given the additional negative impact of diabetes for individuals with already a high risk of cardiovascular disease, recommend screening to begin at age 5years, to ensure it is identified and treated at the earliest stage [9]. Laboratory studies for insulin resistance should include studies of lipids, hepatic function, and glucose homeostasis [7].

### Final thoughts

A majority of the authors of this study have been involved with the national support group for primordial dwarfism for many years. We have been thrilled to see neurovascular screening protocols appear to save lives, to then witness with a heavy heart young adults succumbing to other unanticipated maladies. We are hopeful for the day that we can be a step ahead of all the life-limiting conditions associated with MOPDII. Knowing the landscape is one thing, and knowing how to navigate it is another. We are optimistic this survey is the first step in helping to unravel the key to best take care of individuals with MOPDII.

## Data Availability

The datasets used and/or analyzed during the current study are available from the corresponding author on reasonable request.

## Abbreviations

ACEi: Angiotensin converting enzyme inhibitors
ASD: Atrial septal defect
BB: Beta blockers
BP: Blood pressure
CAD: Coronary artery disease
CCB: Calcium channel blockers
CKD: Chronic kidney disease
CNS: Central nervous system
CT: Computed tomography
CTA: Computed tomography angiography
DASH: Dietary Approaches to Stop Hypertension
ECG: Electrocardiography
HTN: Hypertension
LVH: Left ventricular hypertrophy
MI: Myocardial infarction
MOPDII: Microcephalic osteodysplastic primordial dwarfism type II
MPD: Microcephalic primordial dwarfism
MRA: Magnetic resonance angiography
MRI: Magnetic resonance imaging
NSTEMI: Non-ST-elevation myocardial infarction
OMIM: Online Mendelian inheritance in man
PCNT: Pericentrin
PFO: Patent foramen ovale
STEMI: ST-elevation myocardial infarction
TIA: Transient ischemic attack
VSD: Ventricular septal defect
